# Resident and Family Carer Perspectives on the Impact of Allied Health Student Placements on Service Delivery to Residents in Northern NSW Aged Care Homes: A Qualitative Study

**DOI:** 10.1111/ajr.70160

**Published:** 2026-03-08

**Authors:** Rosie Meares, Mohammad Hamiduzzaman, Vanette McLennan, Sarah Miles, Sarah Crook, Lewis Grove, Frances Barraclough, Jennie Hewitt, Gillian Nisbet, Karn Nelson, Marianne Wallis, Victoria Flood

**Affiliations:** ^1^ Faculty of Medicine and Health University Centre for Rural Health (UCRH), The University of Sydney Lismore New South Wales Australia; ^2^ Whiddon Residential Care Glenfield New South Wales Australia; ^3^ School of Health Sciences, Faculty of Medicine and Health The University of Sydney Sydney New South Wales Australia; ^4^ Faculty of Health Southern Cross University Bilinga Queensland Australia

**Keywords:** access to allied health services, allied health students, Northern NSW, residential aged care, rural clinical placements

## Abstract

**Objective:**

To explore the perceptions of rural residential aged care home (RACH) residents and family carers about the integration of allied health students in service delivery, and its impact on residents' health and wellbeing.

**Setting:**

Two rural RACHs in Northern NSW, where allied health students (Physiotherapy, Occupational Therapy, Speech Pathology, Nutrition and Dietetics and Social Work) attended rural clinical placements and were integrated in service delivery for residents.

**Participants:**

Twelve residents who received student‐integrated services from the RACHs and five family carers.

**Design:**

This was a qualitative evaluation. Residents and family carers were approached to participate in a semi‐structured interview. Interviews were recorded, and audio interviews were transcribed verbatim. The transcripts were analysed using reflexive thematic analysis to generate themes and sub‐themes.

**Results:**

Three themes, each with several sub‐themes, emerged from the interview data. The first theme was facilitators of effective care, and sub‐themes were: modes of services, team‐based approach and continuity of care. Continuity of care between successive groups of students was identified as an aspect of placement that requires improvement. The second theme presented how residents' functional abilities and emotional wellbeing improved after participating in student‐led assessments and interventions. The third theme was participants' satisfaction with the students' competency and professionalism in delivering services.

**Conclusion:**

From the perspectives of residents and family carers, allied health student placements can enhance service provision in rural RACHs in Northern NSW. These placements offer potential to grow the rural allied health workforce and assist in meeting the health service needs of older rural adults within the region.

## Abstract (highlights)

**What Is Already Known on This Subject?:**

- Rural clinical placements create opportunities to address healthcare gaps in communities.
- The benefits of student immersion in healthcare settings are evident for both students and staff.
- Rural clinical placements increase people's access to allied health services.

**What Does This Study Add?:**

- Residents access services from allied health students on placement in rural RACHs, resulting in improved functional independence and emotional wellbeing.
- Multimodal service delivery and multidisciplinary approach are key aspects of student‐assisted services in rural RACHs.
- Continuity of care was identified as a critical component within student‐integrated allied health service provision requiring attention.

## 1 Introduction

Residential aged care homes (RACHs) provide permanent care for those who cannot live independently at home [1, 2, 3]. In 2024, more than 180 000 Australians were in permanent residential aged care [4]. However, the Royal Commission into Aged Care Quality and Safety (2021) reported that RACH residents in rural communities have limited access to allied health professionals—such as physiotherapists, occupational therapists, dietitians and speech pathologists—who are integral to the management of functional independence [5]. The full‐time equivalent number of allied health professionals relative to the population is the lowest in rural communities for all professions [6], requiring an additional 41 286 allied health workers to bring these community Aged‐Care‐Planning Region (ACPR) to parity with the provision in metropolitan ACPR [7, 8, 9]. These communities also experience higher rates of workforce turnover compared to major cities. Limited workforce capacity remains a significant barrier to adequate access to allied health services in RACHs, as reported in the literature [10, 11].

A potential strategy for increasing older rural adults' access to allied health services is through place‐based student placements in RACHs [12, 13, 14]. Rural clinical placements have been shown to improve aged care residents' access to allied health services [15, 16, 17], and to potentially contribute to the growth of the rural healthcare workforce, as students who attend placements in rural areas may be more likely to work rurally after graduation [12, 13, 14]. In 2021, the University Centre for Rural Health (UCRH), in collaboration with other University and residential aged care provider partners received funding from the Commonwealth Government's Rural Health Multidisciplinary Training (RHMT) Aged Care Expansion Programme to develop such placements in Northern NSW. The UCRH placement model has been co‐designed with key stakeholders (i.e., universities, residential aged care homes and allied health professional bodies) and locally contextualised for strengthening aged care services in Northern NSW.

The impact of student‐integrated care provided through rural clinical placements is reported to increase access to allied health services [18, 19, 20, 21, 22, 23]. Studies that evaluated RACH‐based interprofessional education programmes for residents in South Australia, Western Australia and Tasmania found that residents appreciated the additional care time they received from students, as the students' services were not as time‐pressured as those of regular care staff [18, 19]. Research has also shown that interprofessional placements lead to positive health and wellbeing outcomes for aged care residents [23].

Physical health benefits include improved strength, balance, mobility and confidence in completing independent activities of daily living after a 15‐week student‐led physiotherapy programme [21]. Psychosocial benefits include multi‐generational interactions, increased community connections and new perspectives on participating in health building activities [20]. Residents in some studies also reported feeling purposeful by contributing to student learning [19, 20, 21, 22, 23].

Recent waves of aged‐care reforms (2012 Living Longer Living Better (LLLB) initiative and 2019 Australian Royal Commission into Aged Care Quality and Safety) focused on consumer‐directed care and quality of residential care, and led to documentation of outcome measures in Aged Care Quality Standards [11]. Current evaluations of rural clinical placements are centred on student learning and residents' access to healthcare, but lack alignment with relevant Aged Care Quality Standards (i.e., consumer dignity and choice, assessment and planning, personal care and clinical care, services and supports for daily living and service environment) [24, 25]. Additionally, impact of such placements was occasionally explored from consumer perspectives [18, 19, 20, 23], limiting these evaluations and offering little or no guidance for aged‐care partners on how to integrate allied health students in existing care models. This present study aims to answer two questions: what are the perceptions of residents and family carers regarding the integration of allied health students into service delivery for residents? and how do residents and family carers perceive the impact of student placements on residents' health and wellbeing? Answers to these questions from consumer perspectives will help identify how student placement programmes can be optimised to benefit RACH residents.

## 2 Methods

### 2.1 Ethics Statement

This project was approved by the University of Sydney Human Research Ethics Committee (Project identifier: 2023/HE000780).

### 2.2 Study Design

A qualitative evaluation was conducted, involving semi‐structured interviews with residents and family carers and a reflexive thematic analysis of data.

### 2.3 UCRH Placements

The UCRH aged‐care rural clinical placement model and its components have been reported elsewhere [26]. Placements generally ran for 5–20 weeks (occupational therapy: 7–10 weeks, physiotherapy: 5 weeks; nutrition and dietetics: 6 weeks; speech pathology: 6–8 weeks and social work: 20 weeks) and included group and individual care delivery to residents. Students are integrated into service delivery such as conducting clinical assessments, developing care plans, delivering therapeutic interventions, and supporting residents' rehabilitation. They are also involved in multidisciplinary case discussions and team‐based care activities where they work with students from other disciplines, mimicking a multidisciplinary care team approach. The students receive on‐site multidisciplinary clinical supervision and on‐demand online supervision throughout their placements.

### 2.4 Settings and Participants

Residents and family carers who participated in this study were from two rural RACHs in Northern NSW, classified as medium rural town (MM4) and small rural town (MM5). On‐site nurse managers assessed residents' physical and psychological abilities for participation and residents were excluded if they had moderate to advanced dementia or disabilities that prevented them from sharing their insights. Nurse managers then shared the lists of residents (*n* = 22) with the research team. Family carers (*n* = 22) of the residents were also invited to participate.

### 2.5 Data Collection and Dataset Generation

Data were collected from September 2024 to April 2025. In 2024, 52 allied health students completed 334 weeks of placements in two RACHs. Eligible residents were approached by a UCRH research academic with an invitation to be interviewed. Family carers received an online expression of interest form from the marketing and communication department of the RACHs, including a Participation Information Statement. Those who expressed interest were asked to nominate a time and location for the interview. Participation was voluntary, and written or verbal consent was obtained before the interview commenced. All resident interviews were conducted face‐to‐face at the RACHs, while interviews with family carers were conducted either face‐to‐face at the UCRH or via telephone. Participants were interviewed by a research team member, an academic at the UCRH and the project manager for the study, who was not involved in student supervision, assessment or resident care. The interviewer (male) is currently working as a research academic, has a PhD in nursing (aged‐care) and qualitative research, and is experienced in conducting interviews with consumers and service providers. Interviews followed a six‐question semi‐structured interview guide (research team designed and reviewed by educators and health practitioners), which focused on how students were integrated and how students had contributed to improving quality of service delivery and residents' health outcomes (See File S1). Interview duration ranged from 8 to 28 min. The audio‐recorded interviews were transcribed by an external professional transcription provider. Participants were given the opportunity to review their interview transcripts by sharing their contact details during consent; however, no participants requested this, and therefore, no changes to the data were made as a result of member checking.

### 2.6 Data Analysis

Reflexive thematic analysis [27], a six‐step qualitative data analysis method, was used to analyse data because it facilitates the development of nuanced and context‐sensitive themes to gain a deeper understanding. After repetitive reading of the transcripts for data familiarisation, initial coding of transcripts was conducted in NVivo 14. Two interview transcripts were coded by two independent researchers to check for coding consistency. Codes were then categorised and presented in weekly supervision team meetings. Initial sub‐themes and themes were revised following team discussion and presented to the project's investigator team and advisory group members for review. Themes and sub‐themes were finalised based on group feedback.

The COREQ checklist was used to report the findings (See File S2) [28].

## 3 Results

### 3.1 Demographics

Twelve residents (response rate: 55%) and five family carers (response rate: 23%) participated in interviews. There were 10 female and two male residents. All family carers who participated in this study were female, either daughters or partners of the residents.

### 3.2 Data Saturation

Data saturation is important in qualitative research because it indicates the point during analysis at which additional interviews are unlikely to generate materially new themes relevant to our study questions. Consistent with Guest, Namey and Chen's approach [29], we assessed saturation retrospectively using a new information threshold method, selected to provide a transparent and reproducible account of sampling adequacy. In this method, a base size (i.e., a pre‐specified initial set of interviews) is first analysed to establish the set of unique codes; the total number of unique codes in this base forms the denominator. Subsequent interviews are examined in consecutive runs of fixed length; run length refers to the number of consecutive interviews grouped together each time new information is recalculated. For each run, the analyst counts the number of new codes (i.e., codes not previously identified in the base), and computes the percentage of new information as the saturation ratio (See Table 1) [29]:

$$\mathrm{New}\;\text{Information Percentage}=\frac{\text{Total Codes in Base Size}}{\text{Number of}\;\mathrm{New}\;\text{Codes in}\;\mathrm{Run}\;\text{Length}}\times 100\%$$

Saturation is indicated when the percentage of new information falls at or below the pre‐defined threshold (commonly ≤ 5%), suggesting that further interviewing is unlikely to meaningfully expand the thematic code set. For family carers, the number of new codes declined from 22 in FC1 to 0 by FC5 (Table 1). For residents, new codes decreased from 24 in R1 to low levels in later interviews (e.g., 1–2 new codes per interview from R8–R12) (Table 1).

**TABLE 1:** Data saturation for interviews.

Family carer (FC) interview	FC1	FC2	FC3	FC4	FC5
New codes/interview	22	4	2	1	0
Base size: (FC1 + FC2 + FC3) 28 codes		Run length: 2 (FC4 + FC5) – 1 new code		New information threshold: 3.57%	

Resident (R) interview	R1	R2	R3	R4	R5	R6	R7	R8	R9	R10	R11	R12
New codes/interview	24	6	3	2	2	3	2	1	1	2	1	1
Base size: (R1–R10) 46 codes		Run length: 2 (R11 + R12) – 2 new codes					New information threshold: 4.35%					

### 3.3 Identified Themes

Three main themes were identified within the data: (a) Facilitators of effective student integration, (b) health and wellbeing outcomes for residents after participating in student‐led programmes, and (c) participant satisfaction with student‐integrated care.

#### 3.3.1 Theme 1: Facilitators of Effective Student Integration

This theme explores the mechanisms of student integration into RACHs through placement programmes that participants felt enabled students to provide the best care. Three sub‐themes have been identified: modes of care, team‐based approach and continuity of care.

##### 3.3.1.1 Modes of Services

Students provided care to the residents in two models: one‐on‐one and group activities. Participants identified elements of these care models that residents most benefited from. Participants reported that one‐on‐one care facilitated more personalised care. For example, one family carer discussed the involvement of students in their family member's care in ‘setting (my family member) up with an app… for communication’ (FC2) to trial an assistive technology for their family member, who had experienced a stroke, which inhibited their ability to communicate. Another resident described how they benefitted from the individualised physiotherapy sessions, saying, ‘I like the one‐on‐one physiotherapy because that's where I really benefit, especially with my hands now’ (R1). Participants also felt that one‐on‐one care allowed residents to build strong relationships with students, as was expressed by a family carer who said, ‘I just feel [my family member] is feeling much happier now that she's got someone that she's sort of connecting to and goes to the things with them’ (FC4).

The participants also reflected on how the group sessions were beneficial to resident care. Group activities maximised the number of residents that students were able to interact with each week. One resident described the exercise benefits of the walking group for their fitness, saying, ‘we used to go about 9:00, 9:30 and walk around and around the garden and that was good exercise’ (R4), while another shared, ‘Yes, so I go to exercises twice a week and that does me’ (R6). Group therapy also encouraged socialisation between the residents, as was described by a family carer who said, ‘they had a group go together and they used to talk about the past and everyone took the turns about talking about what they used to do when they were… young’ (FC1). However, some participants found that their own physical limitations were a barrier to participating in group sessions. One resident said, ‘I don't stay that long in the activities, because of my mobility [issues] and sore back’ (R9).

##### 3.3.1.2 Team Based Approach

Students collaborated with multiple parties (e.g., students from own and other disciplines, nursing staff, personal care workers, family carers and health professionals) during their placements. Participants reported that this team‐based approach allowed them to understand the residents better and provide more holistic care. The students collaborated with each other to develop care plans, as was described by a resident who recalled, ‘if they gave me one activity, they'd say, “do you think we could do a bit more than that?” And the other one'd say, “perhaps you could try this”’ (R7). They also worked with other disciplines to mimic a multidisciplinary care team often found in the workplace. Another resident said, ‘one particular bloke came here that was bedridden, and between the OTs and the physios, they had him walking all around the place with no aids’ (R5).

Students also met with family carers during their placements to get to know the residents, to report back on their interventions, and to hear family carers' feedback. A family carer described their early interaction with students by saying, ‘I'm sort of telling them what Mum is about and how she reacts and how she acts… so they have a bit more of an understanding when they're trying to talk to her, how to communicate better with her’ (FC1). Family carers expressed that engagement with the students allowed them to encourage and motivate their residents, as they knew what activities the students and residents were doing together. One family carer said, ‘that gives us something else to talk about and to give them positive reinforcement on what she's doing’ (FC4). Another family carer spoke of what happened when they weren't able to be as involved with the students as they would have liked. They said, ‘For example, he didn't want…to use special cutlery. ….so he learnt how to use normal cutlery so he could feel normal. But as soon as the students or anyone goes in, they think, oh, yes, he's got hands like this, he needs special cutlery. But that doesn't work with him’ (FC2).

##### 3.3.1.3 Continuity of Care

Participants identified continuity of care as an important element of student placements, which facilitated impactful care. Consistent student visits enabled the residents to establish a routine that kept them active and motivated, allowing time to build rapport with the students. One resident reported, ‘we all missed [the exercise classes] when we didn't have it, you know, because we needed to just to keep active’ (R4). A family carer suggested that a permanent student position could be established in the RACHs to increase consistency of student interactions and allow students to see more residents as they ‘only come in two days a week and I don't know how many people they have to see’ (FC4).

Participants reported that the short placement blocks and transitions between student groups could disrupt the continuity of care for residents and negatively impact student care delivery. They felt that improvements to residents' health, as well as valuable insights about residents, were lost between student groups. A resident expressed the loss of routine due to this handover, saying, ‘when one lot went, they were supposed to keep on with what we were doing…they didn't always do that though’ (R4). Another family carer noticed how residents were impacted by having to rebuild relationships with new students, stating, ‘by the time [the students] have built up a rapport with the patients they're sort of moving on’ (FC1). They suggested that transition may be smoother if students were able to better share information they had about the residents to incoming students, such as ‘what the resident's favorite things are or what gets them to laugh’ (FC1). Family carers also pondered how constant student transition may require more from busy staff when new students are ‘having to be looked after and supervised’ (FC5) while they learn their role. However, participants felt that the constant introduction of new students could be beneficial because it exposed the residents to new people. A resident noted that students brought ‘fresh thoughts and fresh approaches to care’ (R2). A family carer found that having new students around evoked different memories for their resident, which might ‘trigger her to remember something that she's forgotten in the past’ (FC1).

#### 3.3.2 Theme 2: Health and Wellbeing Outcomes

This theme explores resident and family carer perceptions of the impact of student integration on residents' health and wellbeing, following participation in student‐led therapies. Two sub‐themes related to physical and psychological health outcomes have been identified.

##### 3.3.2.1 Physical Health Outcomes

Participants reported improvements in residents' physical health measures across a range of domains under the guidance of the students. Residents reportedly regained functional abilities, which allowed them to complete more daily tasks and with greater independence. These functional improvements included improved balance, safe movement, speech, walking and overall strength. One resident reflected on their improved mobility, saying, ‘I've learnt to walk again with my walker, and learnt to stand tall’ (R1). Another resident noted that without the student exercise group, ‘We wouldn't have the strength that we learned to build up [in] our bodies’ (R12). A family carer noticed an improvement in her resident's communication ability after working with speech pathology students, saying, ‘his voice wasn't as strong as it should be because he wasn't breathing enough’ (FC2).

Participants also spoke of improvements in existing health conditions. One resident spoke of how the physiotherapy student exercises had helped them manage their arthritis, saying, ‘It's improved my wrist…I can do a whole octave on the piano with that hand now’ (R1). The resident also described having struggled with frozen shoulder but noted it was ‘almost better’ (R1) after following the students' exercise programme.

##### 3.3.2.2 Psychological Health Outcomes

Participants noticed improvements in residents' psychological health outcomes with the integration of student placements. Family carers noticed an improvement in their family members' overall moods. One said, ‘his whole attitude changed’ (FC2) after their family member began interacting regularly with the students. One of the residents imparted how the challenging activities set by the students made them feel accomplished, saying, ‘It sets challenges for us…and makes us feel better’ (R4). Residents described benefits to their mental health through increased engagement and motivation to improve their physical health. One resident said, ‘once the students showed that you were getting somewhere, yeah, you became very motivated’ (R5).

Residents were also noted to have formed emotional attachments to the students. This was interpreted as both a psychological benefit because of the new relationships residents were forming, as well as a challenge because the residents missed the students when they left. A family carer spoke of the relationships they noticed between students and residents when they said, ‘We were talking to the OT one day out there, and other people were going past and she was saying hello. They'll say hello as they're coming past and call her by her name and smile’ (FC4). A resident expressed their sadness when the students left after forming a close bond with them, saying, ‘We do miss them’ (R4), while another stated, ‘You just get used to them, and they go’ (R1).

#### 3.3.3 Theme 3: Satisfaction With Student‐Integrated Care

This theme explores participants' perspectives on how student placements impacted the care given to residents. Two sub‐themes were identified: quality of care and student performance.

##### 3.3.3.1 Quality of Care

Participants felt that students enhanced existing care and provided them with services that were otherwise inaccessible to them. One of the residents remarked on the enhanced person‐centred care provided by students, saying, ‘they keep us safe, and they really care about you’ (R1). Residents also noted how ‘there's been more care’ (R5) due to students being able to spend more time with them than busy RACH staff often could. A resident spoke highly of how often the students were able to interact with residents, saying ‘a couple of ladies that think they're great because [the students] are getting them to walk. They take them for walks all the time’ (R6). Participants felt that the students brought a particular set of skills that they would otherwise not be able to access in the RACH due to economic and logistical restraints. This was highlighted by a resident who said, ‘There is no way that the people could afford the amount of care they are getting through the students …It's not available through what aged care providers give to the people’ (R5).

##### 3.3.3.2 Student Performance

Participants commended the students' conduct when interacting with family carers and residents. They found that the students were very compassionate towards the residents. One family carer found the students to be ‘reassuring’, saying, ‘at least I know she's safe and there's people, caring people looking out and trying to get Mum to remember about her past’ (FC1). Participants also found the students to be competent and professional when interacting with the residents. One resident noted that the students were ‘polite and seemed to know what they were doing’ (R7), while another described them as ‘very thorough’ (R12). A family carer observed how the students interacted with her resident and commented that they had ‘wonderful interpersonal skills’ and ‘communicate with him really well’ (FC2). Another resident emphasised the competency of the students by saying ‘they were very efficient’ but that ‘it wasn't just a five‐minute turnout’ (R7), which indicated the students' ability to provide safe and satisfactory care without wasting time.

## 4 Discussion

This qualitative evaluation investigated consumers' perceptions of the integration of allied health students in care delivery for residents in two rural RACHs in Northern NSW. Residents and family carers in our study had positive perceptions towards this model of service provision and its contributions to residents' health and wellbeing, strengthening current evidence [24, 25, 30, 31]. Participants identified multiple care modes (one‐on‐one and group sessions) and the multidisciplinary approach of the UCRH placements as two important mechanisms for benefiting resident care. They discussed how increased continuity of care between student groups could improve the quality of care. Participants noticed improved physical health outcomes for residents after engaging with student‐led therapies, including better balance, mobility, strength and personal independence. Participants also reported residents' improved moods and increased motivation to maintain their health. However, they reflected that residents often missed students when the placements ended. Both residents and family carers were impressed with students' professional behaviour and the high quality of services provided to residents in a setting where allied health services are otherwise limited. The factors found to facilitate impactful student‐integrated service provision—specifically, continuity of care and a multidisciplinary team approach—are closely aligned with the Aged Care Quality Standards [11].

The need to consider how student integration affects continuity of care for residents was an important aspect identified by our participants, as well as those in previous studies. Seaman, Bulsara and Saunders found that when students left the RACH at the conclusion of their placements, the RACHs struggled with the transition between student groups [20]. Participants in our study reiterated this finding and highlighted the limitations of student placements in maintaining continuity of care for residents, noting that such limitations may undermine residents' ability to exercise choice and access safe services [11]. While mechanisms that facilitate good student learning and mechanisms that enhance resident care may not always be aligned [31, 32, 33], and while university schedules usually dictate the length of placements [34], it is important to consider the impact of frequently transitioning student groups on residents, the primary affected stakeholders. This is especially important in rural areas, where retention rates of aged care workers are lower compared to those in urban areas, and where residents are more likely to experience a high turnover rate of nursing staff and limited or no access to allied health workers [9]. However, despite noting the impact of student transition on continuity of care, student integration was identified as beneficial for all parties [34].

Existing literature has highlighted the benefits of student‐integrated services in improving the physical and psychological health outcomes of older adults [24, 25]. The UCRH aged‐care rural clinical placements integrated allied health students in clinical assessments, care plan development, and interventions/therapies for residents, addressing three quality indicators (assessment and planning, clinical care and support services for daily living). The associated benefits of UCRH placements are supported and expanded upon in literature [24, 25]. Similarly to Seaman and colleagues [20], and Hams and colleagues [21], participants in our study reported an improvement in residents' mobility, balance, and independence after working with students. Participants in our study also found that experiencing physical improvements gave them a sense of achievement and motivation to continue participating in student‐delivered one‐to‐one and group sessions. Seaman and colleagues previously reported on residents enjoying the youthful presence of students [20], a finding which was reflected by our participants who enjoyed the ‘fresh’ approaches brought by new students. Notably, many traditional rural clinical placements have confined students to roles in need assessments and referral, with limited active therapy involvement. By contrast, the UCRH placements tasked students with delivering hands‐on interventions and therapies, which expanded the scope and safety in residential care [24, 35, 36]. Such findings prompt discussion about the optimal integration of students—whether through dedicated student‐led services or by embedding students within existing residential care teams and workflows.

Participants in our study noted the benefits of the multidisciplinary team approach, a sentiment rarely found in existing literature for rural allied health student placements. Participants felt that having students from multiple allied health disciplines working together with one resident enhanced the care provided, as students suggested improvements to each other's care plans. They also found that the involvement of family carers was beneficial because it facilitated effective person‐centred care for residents and strengthened the connections between students and residents. The UCRH multidisciplinary placement model mimics the way that healthcare is moving towards a more holistic, integrated, and person‐centred approach, particularly in acute care settings [37, 38]. It makes sense to continue this care model in RACHs, as many residents have chronic conditions that would typically require concurrent management by multiple allied health professionals in the community [1, 2]. Multidisciplinary collaboration is also beneficial for student learning because it mirrors the healthcare teams that students may be working with in their future placements and career.

In the context of the allied health workforce shortage in rural areas [4, 5], and the Royal Commission into Aged Care Quality and Safety revealing inadequate allied health services in RACHs [8], the satisfaction with student‐integrated care reported in our study and supported by existing literature [19, 22, 23]. Such findings highlight the potential for student‐integration to increase accessibility of allied health services for RACH residents while encouraging allied health workforce development in rural RACHs. Students who undertake placements in rural areas are also more likely to choose to work in rural areas in the future, meeting the quality standard of recruiting a qualified and skilled workforce [12, 13, 14]. Therefore, it is hoped that the impact of the UCRH aged care rural clinical placements may extend beyond the immediate benefit of students providing care to residents to building the allied health workforce in the region [39].

This study has both strengths and limitations. Employing reflexive thematic analysis to generate themes and sub‐themes from residents' and family carers' perspectives generates evidence how to integrate students in residential care and achieve best outcomes from the student‐integrated care [27]. Having an Aged Care Advisory Group, comprising key stakeholders, enabled insights and feedback that identify discussion points and future directions. In our study, a selection bias exists because of participants being able to choose to be interviewed. This may have affected certain family carers, who were not able to take part due to their busy schedules, and a few residents, who had chronic conditions that restricted their participation. This study was also limited by the breadth of participant experiences reported, as only two RACHs in two rural towns were involved, and five family carers participated in interviews. Furthermore, the qualitative method, though effective in capturing resident and family member perceptions and experiences, made it difficult to objectively confirm the contributions of student‐integrated services to residents' health and wellbeing outcomes. A study combining objective measurements of health outcomes with self‐reported measures is recommended for future research.

## 5 Conclusion

This study strengthened and contributed to the limited literature around the effectiveness of allied health student placements in rural RACHs, from residents' and family carers' perspectives. We found that both participant groups had positive perceptions of student‐integrated care, believing that quality of care was enhanced by the UCRH placements, which incorporated multimodal, multidisciplinary, and consistent care approaches. While this study supports the potential of student‐integrated care to contribute to the growth of the rural allied health workforce, rigorous research is required to evaluate the effectiveness of UCRH placements and similar models as they are implemented in other rural areas of Australia.

## Author Contributions

**Rosie Meares:** conceptualization, investigation, funding acquisition, writing – review and editing, validation. **Mohammad Hamiduzzaman:** conceptualization, investigation, writing – original draft, funding acquisition, methodology, validation, visualization, writing – review and editing, software, formal analysis, project administration, data curation, supervision, resources. **Vanette McLennan:** conceptualization, investigation, funding acquisition, writing – review and editing, methodology, validation, visualization, project administration, supervision, resources. **Sarah Miles:** conceptualization, investigation, funding acquisition, writing – review and editing, validation, supervision. **Sarah Crook:** conceptualization, investigation, methodology, writing – review and editing, validation, visualization. **Lewis Grove:** conceptualization, investigation, methodology, writing – review and editing, validation. **Frances Barraclough:** conceptualization, formal analysis, methodology, software, visualization, writing – original draft. **Jennie Hewitt:** conceptualization, investigation, methodology, validation, writing – review and editing. **Gillian Nisbet:** conceptualization, investigation, methodology, writing – review and editing, validation. **Karn Nelson:** conceptualization, investigation, methodology, writing – review and editing, validation. **Marianne Wallis:** conceptualization, investigation, methodology, validation, writing – review and editing. **Victoria Flood:** conceptualization, investigation, funding acquisition, methodology, validation, visualization, writing – review and editing, project administration, supervision, resources.

## Funding

This study was supported by the Rural Health Multidisciplinary Training (RHMT) Aged Care Expansion Grant.

## Conflicts of Interest

The authors declare no conflicts of interest.

## Supporting information


**File S1:** Interview guide for resident/family.

**File S2:** COREQ (COnsolidated criteria for REporting Qualitative research) checklist.

## Data Availability

The data that support the findings of this study are available from the corresponding author upon reasonable request.
