# Supplementary material for: Resident and Family Carer Perspectives on the Impact of Allied Health Student Placements on Service Delivery to Residents in Northern NSW Aged Care Homes: A Qualitative Study
Source: Aust J Rural Health. 2026 Mar 8;34(2):e70160. doi: 10.1111/ajr.70160 (PMC12968508; doi:10.1111/ajr.70160)
Supplement: Supplementary file 1 — File S1: Interview guide for resident/family. [file AJR-34-0-s002.docx]

**Supplementary File 1:** Interview Guide for Resident/Family

**Manuscript Title:** Resident and family carer perspectives on the impact of allied health student placements on the quality of care for residents in Northern NSW aged care homes: A qualitative study

Interview Guide for Resident/Family

This interview will be audiotaped with participant consent and transcribed for analysis and reporting purposes. The following points/questions are provided as a guide for the interviews. The in-depth discussion will be limited to these points/questions unless new factors and issues raised by the participants.

Welcome – Introduction and consent.

1. Have you noticed the allied health/nursing students who have been working with the regular staff?
2. If yes, can you tell me what they have done with you (or the person under your care)
3. What did they help you with? How did the activity go? Can you give me an example of something you did with a student?
4. What do you see as the benefits of student placements in aged care settings?
5. To how the services are delivered? If so, please give me example.
6. To the resident (or your family member) care? If so, please give me an example.
7. What do you see as the drawbacks or challenges of this program?
8. To how the services are delivered? If so, please give me example.
9. To the resident (older adult) care? If so, please give me an example.
10. Do you have any suggestions for addressing the challenges?
11. How would you describe the contribution of the placements to aged care workforce capacity building in rural areas of Northern NSW? What made it work or not work?
12. Do you have any suggestions for the improvement of the placements? Would like to see these placements continue? Why or why not?

I’ve come to the end of our questions. Let me say thank you for your honest opinions. I really appreciate your help.
